# Barriers and facilitators to implementation of proper limb positioning in hemiplegic stroke patients: a qualitative study

**DOI:** 10.1590/1980-220X-REEUSP-2025-0110en

**Published:** 2025-09-15

**Authors:** Xin-Yue Zhang, Min-Min Leng, Jia-Wei Yang, Shuo-Tao Li, Min Li, Xiao-Xu Ji, Quan-Qin Cui, Fang Liang, Xia Chen, Li-Juan Yang

**Affiliations:** 1 Shandong First Medical University, Shandong Provincial Hospital, Department of Neurology, Jinan, China.; 2 Shandong First Medical University, Shandong Provincial Hospital, Department of Nursing, Jinan, China.; 3 Shandong University of Traditional Chinese Medicine, Department of Nursing, Jinan, China.

**Keywords:** Stroke, Nurse Clinicians, Patient Positioning, Qualitative Research, Implementation Science, Acidente Vascular Cerebral, Enfermeiros Clínicos, Posicionamento do Paciente, Pesquisa qualitativa, Ciência da Implementação

## Abstract

**Objective:**

To identify factors that can be targeted to promote the standardized management of proper limb positioning and improve the rehabilitation outcomes of patient.

**Method:**

A qualitative descriptive study was conducted using semi-structured face-to-face in-depth interviews with 24 clinical nurses working in stroke-related departments between April and May 2024. Based on the COM-B model, the data were analyzed by employing the deductive content analysis method using the NVivo12 software and manual coding.

**Results:**

Analysis revealed 11 themes and 22 sub-themes associated with the four domains of the COM-B model, including 15 barriers and 7 facilitators.

**Conclusion:**

Clinical nurses&apos; adherence to proper limb positioning is dynamic and positioning should address barriers related to capability, opportunity, and motivation, such as prioritize knowledge and skills training for clinical nurses, improved compliance among patients and caregivers, establish robust systems and supervisory mechanisms, and support scientific research and innovation to explore intelligent management solutions.

## RESUMO

**Objetivo:**

Identificar fatores que podem ser alvo de intervenções para promover a gestão padronizada do posicionamento adequado dos membros e melhorar os resultados da reabilitação dos pacientes.

**Método:**

Foi realizado um estudo descritivo qualitativo por meio de entrevistas semiestruturadas presenciais com 24 enfermeiros clínicos que atuam em departamentos relacionados ao AVC, entre abril e maio de 2024. Com base no modelo COM-B, os dados foram analisados utilizando o método de análise dedutiva de conteúdo com o software NVivo12 e codificação manual.

**Resultados:**

A análise revelou 11 temas e 22 subtemas associados aos quatro domínios do modelo COM-B, incluindo 15 barreiras e 7 facilitadores.

**Conclusão:**

A adesão dos enfermeiros clínicos ao posicionamento adequado dos membros é dinâmica, e o posicionamento deve abordar barreiras relacionadas à capacidade, oportunidade e motivação, tais como priorizar o treinamento de conhecimento e habilidades para os enfermeiros clínicos, melhorar a adesão dos pacientes e cuidadores, estabelecer sistemas e mecanismos robustos de supervisão, e apoiar a pesquisa científica e inovação para explorar soluções inteligentes de gestão.

## INTRODUCTION

Stroke is a significant public health problem globally and a leading cause of long-term disability and mortality^(1)^. It is projected that over 11.9 million people worldwide suffer from stroke, with an estimated 7.3 million deaths. Additionally, stroke accounts for 5.6% of all edisability-adjusted life years (DALY)^(2)^. Globally, China has the highest lifetime risk of stroke, which has become the leading cause of disability and death among adults^(2)^. Data indicate that approximately 3 million new stroke cases occur in China each year, with over 80% of these patients experiencing varying degrees of dysfunction, the most common manifestation being limb hemiplegia^(3)^, which makes patients life independence, life quality and social adaptability decreased significantly while also imposing significant mental stress and economic burdens on individuals, families, and society^(4)^. The timely, scientific, and effective implementation of rehabilitation treatment to improve recovery outcomes for stroke patients remains a critical focus of researchers and healthcare professionals worldwide.

Early rehabilitation is the most effective method for reducing the disability rate following a stroke, as confirmed by evidence-based medicine^(5,6)^. Proper limb positioning as a temporary position to prevent or combat spasticity is a key component of early rehabilitation and may have a significant impact on public health^(7)^. Several guidelines emphasize the importance of maintaining proper limb positioning in stroke patients^(5,6,8)^. It can indirectly promote the formation and remodeling of nerve synapses, repair the damaged neurons, and is of great value in reducing the complications of patients, accelerating the rehabilitation process, improving the quality of life and reducing the economic burden^(6)^. In addition, the practicality and cost-effectiveness of it are well-documented in clinical guidelines and expert consensus statements^(5,8)^. Despite widespread agreement on its effectiveness, clinical nurses (CNs) have not consistently implemented proper limb positioning in daily practice, potentially hindering the rehabilitation progress of stroke patients^(9)^. Promoting the standardized management of proper limb positioning warrants further investigation.

## BACKGROUND

Some scholars point out that the compliance of CNs may be an important reason to hinder the development of proper limb positioning^(7,10)^, and it is necessary to clarify the influencing factors of their implementation of proper limb positioning. Previous studies have found that the implementation rate of proper limb positioning remains low, possibly due to factors such as marital status, hemiplegic limb lateral separation, bed-to-care-giver ratio, position, system and regulatory strategy^(10,11)^. The cognitive level of stroke patients and caregivers also needs to be improved, which also reflects the lack of education efforts^(12)^. To address these challenges, researchers developed an evidence-based nursing review index based on the Johns Hopkins guidelines. Further, they analyzed the barriers to the implementation of proper limb positioning^(10)^. However, they did not focus on the implementation experiences of healthcare professionals from a qualitative perspective. It is worth noting that effective implementation of proper limb positioning requires the collaboration of healthcare providers, patients, and family members. As primary implementers and educators of proper limb positioning, nurses’ knowledge, attitudes, and experiences can influence adherence among patients and their families^(13)^. Thus, conducting in-depth interviews to explore nurses’ experiences with implementing proper limb positioning can yield deeper, more complex, and more comprehensive insights into their experiences, attitudes, or behaviors.

**Figure 1:**
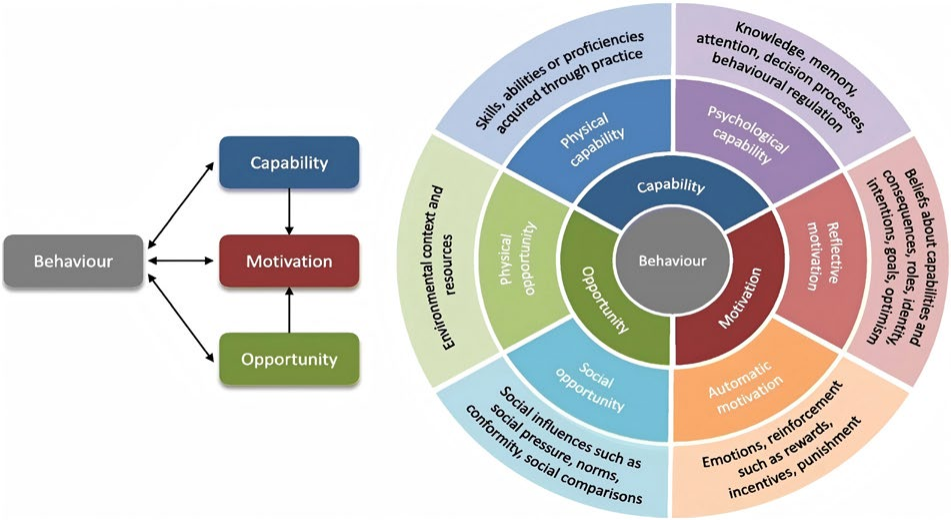
The COM-B model^(15)^.

Research indicates that using a theoretical framework to understand behavior is one of the most effective approaches^(14)^. The Capability, Opportunity, Motivation-Behavior (COM-B) model, first proposed by Michie et al.^(15)^, serves as the core of the behavior change wheel (BCW). This model is designed to identify the target behaviors of specific populations and facilitate behavior change (Figure 1). The COM-B model emphasizes that capability, opportunity, and motivation within a behavioral system interact and influence one another. These interactions are shaped by an individual’s physical and mental capabilities, environmental and social opportunities, and reflective and automatic processes, which ultimately determine behavioral outcomes. Multiple studies have demonstrated that the model effectively identifies barriers and facilitators, providing potential targets for intervention design and serving as a robust framework for health behavior research^(15-17)^.

Therefore, this study applies the COM-B model to conduct a qualitative study exploration of the factors promoting and hindering clinical nurses’ implementation of proper limb positioning. The aim is to identify the influencing factors that can be targeted to facilitate the standardization of limb positioning management and improve the rehabilitation outcomes for stroke patients.

## METHOD

### Design of Study

Given the exploratory nature of the research question, a descriptive phenomenological approach was adopted for this study^(18)^. This methodology is particularly suited to research that captures participants’ experiences, especially in contexts where little is known about the phenomenon being investigated. Study reporting adhered to the Consolidated Criteria for Reporting Qualitative Research (COREQ) (File S1).

### Population

To ensure the representativeness of the sample, we recruited CNs from 9 hospitals in Shandong, China via online recruitment from April to May 2024. Maximum variation sampling, a form of purposive sampling, was employed to select CNs, ensuring comprehensive information and enhancing the external validity of the study(18). The inclusion criteria were: (1) CNs working in stroke-related departments, including neurology, neurosurgery, and rehabilitation; (2) possession of a valid nursing license; and (3) voluntary participation with written consent. The exclusion criteria were: (1) CNs on vacation, transfer, further studies, and regular training; (2) participation in other clinical trials; and (3) withdrawal during the interview process. The sample size was determined by thematic saturation. A total of 27 CNs were invited, 2 declined due to scheduling conflicts, and 1 withdrew during the interview process. Ultimately, interviews with 24 nurses achieved thematic saturation.

### Data Collection

Individual semi-structured face-to-face in-depth interviews were adopted for data collection. The research team developed the semi-structured interview guide by reviewing relevant literature, integrating the COM-B model framework, and expert consultation to finalize the content. To pre-test the interview themes and structure, pilot interviews were conducted with two CNs before the study commenced. Content and comprehensibility of the interview guide appeared to be good. Therefore, no adaptations to the interview guide were made. In addition, we also tested the performance of the voice recorder and found no problems. All interviews were conducted by two postgraduate researchers trained in in-depth interviewing techniques with a professional background in stroke nursing (with prior experience in conducting 15 interviews), whose participation was motivated by clinical observations and research-driven value. Before the formal interviews, the study objectives, methods, and confidentiality protocols were explained to participants. Interviews were recorded in form of audio and notes were made throughout after obtaining the informed consent. To protect the privacy of the participants, the interview was conducted in a quiet and comfortable department room, with no one present except the participants and researchers. Participants’ verbal and non-verbal cues were observed and recorded during the interview. Each session lasted approximately 30 to 45 minutes. Data collection persisted until achieving thematic saturation, operationalized as the absence of novel themes in three successive interviews, upon which participant recruitment was terminated.

### Data Analysis and Treatment

Respondents were assigned identification numbers based on the order of their interviews to protect their privacy. Within 12 hours of each interview, the audio recordings were independently transcribed into text by two researchers and cross-checked to ensure data accuracy. Ambiguities were clarified with the interviewees promptly to maintain the authenticity of the content. The data were analyzed by employing the deductive content analysis method using the NVivo12 software and manual coding^(19)^. The data was prepared and analysis done based on the three antecedents of the COM-B model (capability, opportunities and motivation). The research team collectively developed a coding scheme based on the COM-B model to ensure coding consistency. Following this scheme, two researchers independently conducted the first-round coding of all transcripts, with weekly coding meetings held to discuss discrepancies and reach consensus. Codes were categorized or illustrated within the model’s constructs. If any content did not align with existing codes, a new code would be generated and subsequently evaluated during the revision process. We invited third-party experts to review the coding results. After repeated revisions of the entries and codes, the final categories and themes were summarized from an overall perspective, and the findings were interpreted accordingly.

### Ethical Aspects

This research adheres to the ethical standards outlined in the Declaration of Helsinki and the Dutch Code of Conduct for Scientific Integrity^(20)^. The study protocol was approved by the Ethics Committee (Approval No. SWYX: 2024-029). All participants signed written informed consent, and all data collected were used exclusively for this study.

## RESULTS

A total of 24 registered nurses aged 25 to 47 participated in semi-structured, face-to-face interviews. Most of the nurses worked in tertiary hospital settings (n = 13, 54.2%), and the majority were female (n = 23, 95.8%). Their clinical experience ranged from 1 to 29 years. Details of the nurses’ characteristics are presented in Table 1.

**Table 1:** Participants demographic background – Shandong, China, 2024.

Feature	Overall (n = 24)
Gender	
Man	1 (4.2%)
Woman	23 (95.8%)
Age (x ± s)	34.38 ± 6.96
Hospital level	
Secondary hospital	7 (29.2%)
Tertiary hospital	17 (70.8%)
Department	
Neurology	17 (70.8%)
Department of Neurohealth	3 (12.5%)
Military sursery	2 (8.3%)
Department of rehabilitation medicine	2 (8.3%)
Education background	
Undergraduate course	23 (95.8%)
Postgraduate	1 (4.2%)
Years of working (x ± s)	12.02 ± 7.77
Professional ranks and titles	
Nurse	2 (8.3%)
Primary nurse	8 (33.3%)
Nurse-in-charge	11 (45.8%)
Co-chief superintendent nurse	3 (12.5%)
Position	
Nurse	21 (87.5%)
Head nurse	3 (12.5%)

Clinical nurses (CNs) expressed a strong desire to implement proper limb positioning; however, their compliance was influenced by multiple factors. The analysis revealed 11 themes related to the four domains of the COM-B model and 22 subthemes, including 15 barriers and 7 facilitators. These themes are closely aligned with the core components of the COM-B model and are detailed in Table 2.

**Table 2:** Identified barriers and facilitators in the various domains of the COM-B – Shandong, China, 2024.

COM-B	Themes	Sub-themes	Barriers/Facilitators
Capabilities	The patients’ physical condition	Disease condition	Barriers
		Special patients	Barriers
	Knowledge, skills and accessibility of information	Lack of knowledge and skills	Barriers
		The training effect is not good	Barriers
	Compliance of patient and family	Cognitive levels are limited	Barriers
		Changes in the way of education	Facilitators
		Demographic characteristics	Barriers
Opportunity	Availability of time	Time-consuming operation	Barriers
		High intensity work	Barriers
	Auxiliary means	The scarcity of quantity	Barriers
		Differences in quality	Barriers
	Allocation and utilization of medical and health resources	Unreasonable allocation of human resources	Barriers
		Multidisciplinary collaboration and communication	Facilitators
		The distribution of medical resources is uneven	Barriers
	Social influence	Good working atmosphere	Facilitators
Motivation	The relevant system and supervision strategy	Lack of evaluation criteria	Barriers
		Lack of regulatory strategy	Barriers
	Belief about consequences	The positive effect of proper limb positioning	Facilitators
		Positive feedback	Facilitators
	Guidelines and policies related to proper limb positioning	Guidelines and policies to guide the practice of proper limb are lacking	Barriers
	A sense of professional worth	Work sense of achievement	Facilitators
		Work Ethic	Facilitators

### Capabilities

#### The Patients’ Physical Condition

Many CNs reported that, although willing to implement proper limb positioning, patients often became irritable or uncomfortable, making it difficult to maintain the desired posture. This was sometimes impossible due to high muscle tone and stiff limbs. “The patient is irritable or vague, so he may regain his own state after a while” (CN1).

Additionally, CNs noted that implementing proper limb positioning for patients with large weight or pressure ulcers posed considerable physical, human, and material challenges. “If the patient weighs too much, it will be difficult” (CN12).

Thus, the difficulty of implementing proper limb positioning is significantly increased by patient-specific factors and further exacerbated by limitations in nursing resources.

#### Knowledge, Skills, and Accessibility of Information

Unfortunately, many CNs expressed uncertainty regarding the timing and principles of proper limb positioning. “There is no time, and the principle of placement is not very clear” (CN1). Nurses with less work experience reported greater challenges due to insufficient knowledge. “I am a junior nurse, and I feel a little difficult to contact with it” (CN4).

Conversely, possessing adequate knowledge and skills is a facilitating factor. Therefore, several CNs emphasized the need for improved training methods and better access to information. “It is recommended that the training combine theory and practice so that I will be clearer when I present it to patients next time” (CN10).

Thus, insufficient professional competence restricts the standardized implementation of proper limb positioning, and poor training effect affects the accessibility of information.

#### Compliance of Patient and Family

All CNs emphasized that proper limb positioning requires the cooperation of patients, caregivers, and nurses. However, many patients and caregivers fail to recognize the importance of proper limb positioning and may even express skepticism due to the lack of immediate visible results. “Caregivers don’t understand why they do it because they don’t see any clear difference” (CN1).

Health education plays a vital role in improving the cognitive levels of patients and caregivers. Many CNs reported adopting innovative educational methods, achieving promising results. “We have made a QR code to make the propaganda content into pictures or animated videos, which is more convenient and intuitive” (CN20). “We organize lectures and competitions regularly, and the results are good” (CN15).

The characteristics of patients and caregivers, such as age, education level, and patience, were identified as potential barriers to effective knowledge transmission. This highlights the importance of nurses mastering communication skills to enhance understanding. “I’m tired of teaching old people because they’re hard to learn” (CN11).

Thus it can be seen from this that such as cognitive level, educational methods, and Demographic characteristics affect the degree to which patients and their families cooperate with the treatment.

### Opportunity

#### Availability of Time

The time availability posed a significant challenge. Many CNs noted that maintaining proper limb positioning requires turning patients every two hours. Since the patient often cannot sustain the correct posture, constant supervision and adjustments are needed, which is time- and energy-intensive. “I think the implementation is too complicated. It is impossible to adjust all the time” (CN2).

Additionally, the high intensity of work further exacerbates the challenges faced by CNs. “Stroke patients are more serious, with more nursing operations and basic care” (CN16). Some CNs suggested that integrating intelligent tools or technology could improve the process. “If there is an intelligent method to solve it, the implementation of proper limb positioning is not so troublesome” (CN1).

Thus, the complexity of the operation and the work pressure have intensified the consumption of time and energy. To solve this problem, CNs suggests using intelligent technologies to assist in management.

#### Auxiliary Means

Multiple pillows and other tools are often used to support the patient’s body. However, the lack of adequate auxiliary tools significantly impacts the implementation of proper limb positioning. “Caregivers may not be able to buy pillows; the ward is not so many, can only provide patients with a pillow” (CN24). “I hope the hospital will provide some of the funds and provide more auxiliary tools” (CN8).

Moreover, the quality of the auxiliary tools also affects their effectiveness. Issues such as insufficient support after prolonged use, uncomfortable materials, and the inability to adjust the height or angles pose challenges. “The patient lies down, the pillow cannot reach that height, the turning pillow is a little hard, which is bad for the patient’s skin” (CN23).

Thus, the insufficient quantity and quality defects of assistive tools hinder the implementation of proper limb positioning and may even bring adverse effects to patients.

#### Allocation and Utilization of Medical and Health Resources

Many CNs reported that the unreasonable allocation of human resources poses a significant barrier to proper limb positioning. “There are 80 or 90 patients in our department, but only 15 nurses, so lack of manpower is a problem that cannot be ignored” (CN13).

To address these challenges, some CNs have formed Multi-Disciplinary Treatment (MDT) teams, which reduce the workload and transcended professional boundaries, fostering trust between doctors and patients. “Doctors attach more importance to functional positions and will guide us more” (CN7). “When we speak, it doesn’t carry as much weight as doctors, and patients believe in doctors more” (CN24).

The lack of emphasis on proper limb positioning in primary care hospitals directly impacts patient prognosis, a concern frequently expressed by nurses. “We regret that patients from basic hospitals have foot drops that cannot be corrected” (CN15). To mitigate this issue, nurses suggested strengthening collaboration and communication between hospitals. “I think I need to go to the superior hospital to study, and I can see more” (CN3).

Thus, the shortage of human resources and the insufficiency of grassroots resources have hindered standardized care, while multidisciplinary and inter-hospital cooperation can help alleviate this problem.

#### Social Influence

The social dynamics of individuals and groups can influence the attitudes and behaviors of others in specific directions. Several CNs highlighted that a positive working atmosphere motivates them to implement proper limb positioning. “We communicate well with patients and caregivers, and we are also willing to educate them” (CN19). “We do not do it alone, but the whole department is doing this, so it is easier to implement it.” (CN4).

Thus, positive team culture and doctor-patient communication can positively influence nursing behaviors and reduce the difficulty of implementing proper limb positioning.

### Motivation

#### The Relevant System and Supervision Strategy

Many CNs reported the lack of clinically available evaluation criteria for good limbs, making it difficult to assess the effectiveness of implementation. “Evaluation standard for the proper limb positioning mainly depends on the intuitive functional recovery” (CN18).

Additionally, Several CNs observed that hospitals and departments are not strict in implementing proper limbs. In contrast, leadership attention significantly enhances compliance. “Although inspected twice a week, it does not seem to have been implemented, so supervision of the department can be a little bigger” (CN10).

Thus, the lack of evaluation criteria and regulatory strategy has reduced the emphasis and implementation ability of the CNs on correct limb positioning, which is an important objective factor.

#### Belief about Consequences

All CNs agreed that proper limb positioning positively impacts patients, families, and society while also reducing nurses’ workload to some extent. “Early intervention can promote the recovery of the patient’s limb function and prevent some complications.” (CN4). “If pressure ulcers occur, it will increase our workload” (CN19). From the patient’s perspective, CNs noted that proper limb positioning is a relatively simple and cost-effective intervention. “Teach caregivers how to operate; he can also do it at home” (CN6).

Moreover, positive feedback from patients and caregivers and observable effectiveness further motivate internal drive to make them more convinced of the benefits of proper limb positioning. “Some patients’ limb edema is significantly reduced, indicating the implementation was effective” (CN11).

CNs generally recognizes the win-win benefits of proper limb positioning, and positive feedback further strengthens its belief in the outcome, forming a virtuous cycle.

#### Guidelines and Policies Related to Proper Limb Positioning

Several CNs expressed the desire to adopt more standardized practices but highlighted the absence of authoritative guidelines or expert consensus to guide clinical implementation. “We really want to carry out the proper limb positioning, but the index issued by the state has not been found” (CN3).

Regarding policy, CNs emphasized the need for greater national attention to developing the primary healthcare system. “Let the basic hospitals learn more, improve the awareness of good limb placement” (CN8).

It can be seen from this that the lack of authoritative guidelines and policy support has restricted the professional ability development of CNs and weakened their practical motivation.

#### A Sense of Professional Worth

Several CNs noted that positive patient outcomes reinforce the value and significance of their efforts. “Patients with a long hospital stay, with no contractures or better recovery, will have a great sense of achievement” (CN1).

Additionally, professional ethics play a key role in driving their commitment to proper limb positioning. “The concept and thought have formed the inertia of thinking. If a patient has hemiplegia, you should guide him and help him with functional rehabilitation” (CN8).

Thus, professional accomplishment and work ethics reinforce CNs’ recognition of professional value, which serves as a driving force for standardized nursing practice.

These findings reveal that proper limb positioning implementation faces interconnected patient (physical/cognitive barriers), nurse (knowledge/time constraints), and systemic (resources/training) challenges. While some patient factors are inherent, modifiable barriers like training, health education, and equipment improvements offer actionable solutions, with technology and innovative education showing particular promise. The interdependence of these obstacles emphasizes the necessity of the integrated interventions of capability, opportunity and motivation to drive sustainable behavior change.

## DISCUSSION

This study identified 11 themes that aligned seamlessly with the COM-B model, offering a comprehensive understanding of the factors influencing nurses’ implementation of proper limb positioning^(15)^. The findings suggest that nurses’ adherence to proper limb positioning is a dynamic and balanced process influenced by their capability, opportunity, and motivation.

Solid knowledge and skills were essential for effective practice^(10,12)^. However, in the present study, CNs appeared to view the timing and implementation of proper limb positioning as completely independent problems, with some perceiving them as unrelated to their professional scope. The confidence and experience derived from robust knowledge and skills play a crucial role in promoting the standardization of proper limb positioning^(7,21)^. Accordingly, CNs in this study expressed a strong need for training to acquire knowledge and skills related to proper limb positioning, suggesting that traditional training methods are increasingly inadequate in meeting their learning needs^(22)^. Given the rigor required in medical practice, evidence-based practice education are expected to improve nurses’ attitudes, knowledge, skills, self-efficacy, and professional behavior^(23)^. These interventions should also emphasize the importance of developing comprehensive guidelines and expert consensus on limb positioning. As is highlighted by CNs, even with limited resources, accessing guidelines or expert consensus can enhance their intrinsic motivation, thereby promoting adherence to proper limb positioning practices.

The degree of alignment between the behaviors of patients and caregivers in pursuing recovery and adhering to medical advice significantly inf luences CNs’ attitudes toward their role in patient care, consistent with previous research^(24)^. Understanding the importance of proper limb positioning and its role in recovery is essential for fostering a positive attitude toward decision-making participation^(25)^. This gap in knowledge and trust is a fundamental reason for the skepticism observed among patients and their families in this study. Several guidelines emphasize the need for healthcare professionals to prioritize rehabilitation education for stroke patients^(5,6)^. Although intelligent nursing and health education delivery reforms have succeeded, it still faces multiple challenges such as digital divide, information overload, insufficient personalization, and privacy security^(26)^. In addition to compliance issues, factors such as patient condition, weight, and complications pose challenges to CNs. For patients facing such barriers, it is important to explore optimal strategies for early rehabilitation and proper limb positioning.

Although proper limb positioning offers numerous advantages, its persistence, detailed nature, and tedious processes require substantial time, manpower, material resources, and physical effort^(9,11)^. These demands, particularly in high-intensity work environments, the fragmented, chaotic, and nonlinear nature of nursing work, coupled with the high-quality development of public hospitals and the decreasing average length of hospital stays, complicates CNs’ ability to prioritize patient limb positioning practices^(27)^. The unreasonable allocation of medical resources exacerbates this challenge, such as bed-to-care-giver ratio and the unequal distribution of medical resources between urban and rural areas^(28)^. To address these problems, some researchers have developed human resource allocation models based on nursing hours or case mix indices, established regional medical alliances and multidisciplinary collaborative teams to optimize and integrate medical resources^(29)^. These insights suggest that both the state and hospitals should actively work toward advancing capacity modernization to fundamentally address resource challenges. Furthermore, a positive working atmosphere can encourage CNs to self-regulate and adopt best practices, which is attributed to the fact that individuals’ thoughts, feelings, attitudes and behaviors are often influenced by the words and actions of others(25).

Implementing proper limb positioning is notably influenced by the availability and quality of auxiliary support, which represent significant barriers consistent with the findings from previous studies^(10,11)^. In current clinical practice, caregivers and CNs rely on bedding, pillows, turning pillows, and other tools to assist patients in adjusting. However, issues related to the quantity and quality of these supports often compromise the effectiveness of proper limb positioning^(11)^. The insufficient availability of auxiliary supports may stem from hospital funding constraints, the surrounding environment, and the economic circumstances or purchase intention of patients and caregivers^(9)^. Additionally, the quality of auxiliary support should be considered. The material, size, and angle of auxiliary support significantly influence the effective implementation of proper limb positioning and can even pose health risks to patients^(11)^. Examples include shoulder pain caused by improper limb positioning and skin pressure injuries resulting from sheer force. These highlight the importance of improving auxiliary support systems. Intelligent nursing offers new opportunities for the intelligent management of proper limb positioning. Leveraging artificial intelligence technology to develop intelligent management devices could address the limitations of traditional auxiliary supports, which represents a key area of focus for future research.

Finally, in the domain of motivation, CNs expressed strong recognition and appreciation for the early implementation of proper limb positioning, highlighting it as a key factor in promoting its development^(16,17)^. Meanwhile, positive feedback from patients, caregivers, and implementation outcomes further reinforces their motivation. Additionally, a sense of professional achievement and adherence to ethical principles contribute to their self-efficacy, enabling them to overcome certain challenges in practicing proper limb positioning. However, the motivation is inevitably influenced by objective factors, such as the lack of standardized evaluation criteria and regulatory mechanisms, which may influence behavior adjustment and deviate from expectations^(10)^. Thus, there is a need to improve incentives and sustain the development of CNs behavior.

The examination of sociodemographic data further supports the findings of this study: Advanced CNs (≥10 years of experience, n = 16/24) prioritizes system-level issues, while junior CNs (≤3 years, n = 5/24) focuses on skills, which is consistent with Benner’s novus-expert theory^(30)^. The CNs in tertiary hospitals shows better process familiarity than their counterparts in secondary hospitals, highlighting that differences in training resources can affect capacity building. All these highlight the dynamic interaction of capabilities, opportunity and motivation in behavioral transformation. It should be noted that an insufficient male CNs sample (4.2%) may affect the analysis of gender differences, and future studies will further optimize it.

### Limitations

This study has several limitations. First, phenomenology emphasizes the interpretive role of researchers and may be influenced by their experiences and values, resulting in subjective biases. Second, the small sample size typical of phenomenological studies, while allowing for in-depth exploration, may constrain the generalizability of findings to broader populations. Third, this study was restricted to the perspectives of neurospecialist nurses. Incorporating the perspectives of patients, caregivers, and physicians could provide a broader understanding. Lastly, the transferability of findings to CNs outside China may be limited due to variations in CN roles and responsibilities across countries, necessitating further exploration.

## CONCLUSION

The results indicate that CNs adherence to proper limb positioning is dynamic and positioning should address barriers related to capability, opportunity, and motivation. This underscores the importance of enhanced training for CNs in limb positioning knowledge and skills, as these form the foundation of professional practice. Such training should be complemented by efforts to improve compliance among patients and their families. Additionally, the state and healthcare institutions should prioritize the rational allocation and use of medical resources, which includes optimizing resource allocation, promoting multidisciplinary collaborative models, establishing monitoring and evaluation mechanisms, conducting scientific research and innovation, etc., to standardize proper limb positioning practices and improve patient outcomes.

### Implications

The insights gained from this study can inform the development of tailored training programs and implementation strategies for CN, while also providing direction for the intelligent transformation of proper limb positioning management. By enhancing their professional practice capacity and innovating workflow processes, these initiatives will ultimately improve rehabilitation outcomes and prognoses for stroke patients.

## Data Availability

The full dataset supporting the findings of this study is available upon request to the corresponding author [Xia Chen]. The dataset is not publicly available due to containing information that compromises the privacy of research participants.

## SUPPLEMENTARY MATERIAL

The following online material is available for this article:

File S1 – PRISMA 2020 Checklist.
