# Supplementary material for: Barriers and facilitators to implementation of proper limb positioning in hemiplegic stroke patients: a qualitative study
Source: Rev Esc Enferm USP. 2025 Sep 15;59:e20250110. doi: 10.1590/1980-220X-REEUSP-2025-0110en (PMC12439536; doi:10.1590/1980-220X-REEUSP-2025-0110en)
Supplement: Supplementary file 2 [file 1980-220X-reeusp-59-e20250110-suppl2.pdf]

Rev Esc Enferm USP
https://doi.org/10.1590/1980-220X-REEUSP-2025-0110es

Material Suplementar para “Barreras y facilitadores para la implementación de la correcta posición de las extremidades en pacientes con hemiplejía por accidente cerebrovascular: un estudio cualitativo”

PRISMA 2020 Checklist

Section and Topic	Item #	Checklist item	Location where item is reported
**Domain 1: Research team and reflexivity**			
**Personal Characteristics**			
Interviewer/facilitator	1	Which author/s conducted the interview or focus group?	Page 4
Credentials	2	What were the researcher's credentials? E.g. PhD, MD	Page 4
Occupation	3	What was their occupation at the time of the study?	Page 4
Gender	4	Was the researcher male or female?	Title page
Experience and training	5	What experience or training did the researcher have?	Page 4
**Relationship with participants**			
Relationship established	6	Was a relationship established prior to study commencement?	Page 9
Participant knowledge of the interviewer	7	What did the participants know about the researcher? e.g. personal goals, reasons for doing the research	Page 7
Interviewer characteristics	8	What characteristics were reported about the interviewer/facilitator? e.g. Bias, assumptions, reasons and interests in the research topic	Page 9
**Domain 2: study design**			
**Theoretical framework**			
Methodological orientation and Theory	9	What methodological orientation was stated to underpin the study? e.g. grounded theory, discourse analysis, ethnography, phenomenology, content analysis	Page 5
**Participant selection**			
Sampling	10	How were participants selected? e.g. purposive, convenience, consecutive, snowball	Page 7
Method of approach	11	How were participants approached? e.g. face-to-face, telephone, mail, email	Page 8
Sample size	12	How many participants were in the study?	Page 9
Non-participation	13	How many people refused to participate or dropped out? Reasons?	Page 9
**Setting**			
Setting of data collection	14	Where was the data collected? e.g. home, clinic, workplace	Page 8
Presence of non-participants	15	Was anyone else present besides the participants and researchers?	Page 9
Description of sample	16	What are the important characteristics of the sample? e.g. demographic data, date	Page 10
**Data collection**			
Interview guide	17	Were questions, prompts, guides provided by the authors? Was it pilot tested?	Page 7
Repeat interviews	18	Were repeat interviews carried out? If yes, how many?	No
Audio/visual recording	19	Did the research use audio or visual recording to collect the data?	Page 8
Field notes	20	Were field notes made during and/or after the interview or focus group?	Page 8
Duration	21	What was the duration of the interviews or focus group?	Page 8
Data saturation	22	Was data saturation discussed?	Page 8
Transcripts returned	23	Were transcripts returned to participants for comment and/or correction?	Page 8

Domain 3: analysis and findings			
**Data analysis**			
Number of data coders	24	How many data coders coded the data?	Page 8
Description of the coding tree	25	Did authors provide a description of the coding tree?	Page 8
Derivation of themes	26	Were themes identified in advance or derived from the data?	Page 10
Software	27	What software, if applicable, was used to manage the data?	Page 8
Participant checking	28	Did participants provide feedback on the findings?	Page 11
**Reporting**			
Quotations presented	29	Were participant quotations presented to illustrate the themes / findings? Was each quotation identified? e.g. participant number	Page 12-17
Data and findings consistent	30	Was there consistency between the data presented and the findings?	Page 9-17
Clarity of major themes	31	Were major themes clearly presented in the findings?	Page 12-17
Clarity of minor themes	32	Is there a description of diverse cases or discussion of minor themes?	Page 12-17

From: Tong A, Saisbury P, Craig J. Consoildated criteria for reporting qualitative research (COREQ): 32-item checklist for interviews and focus groups [J]. International Journal for Quality in Health Care, 2007, 19 (6): 349-357.

For more information, visit: http://intqhc.oxfordjournals.org/content/19/6/349.long
