# Supplementary material for: Barriers and facilitators to implementation of proper limb positioning in hemiplegic stroke patients: a qualitative study
Source: Rev Esc Enferm USP. 2025 Sep 15;59:e20250110. doi: 10.1590/1980-220X-REEUSP-2025-0110en (PMC12439536; doi:10.1590/1980-220X-REEUSP-2025-0110en)
Supplement: Translation

# Translation: Barreras y facilitadores para la implementación de la correcta posición de las extremidades en pacientes con hemiplejía por accidente cerebrovascular: un estudio cualitativo

**DOI:** 10.1590/1980-220X-REEUSP-2025-0110es

**Authors:** Xin-Yue Zhang, Min-Min Leng, Jia-Wei Yang, Shuo-Tao Li, Min Li, Xiao-Xu Ji, Quan-Qin Cui, Fang Liang, Xia Chen, Li-Juan Yang

**Affiliations:** 1 Shandong First Medical University, Shandong Provincial Hospital, Department of Neurology, Jinan, China.; 2 Shandong First Medical University, Shandong Provincial Hospital, Department of Nursing, Jinan, China.; 3 Shandong University of Traditional Chinese Medicine, Department of Nursing, Jinan, China.

**Keywords:** Accidente Cerebrovascular, Enfermeras clínicas, Posicionamiento del Paciente, Investigación Cualitativa, Ciencia de la Implementación

## Abstract

**Objetivo:**

Identificar factores que pueden ser abordados para promover la gestión estandarizada de la correcta posición de las extremidades y mejorar los resultados de rehabilitación de los pacientes.

**Método:**

Se realizó un estudio descriptivo cualitativo mediante entrevistas semiestructuradas en profundidad, cara a cara, con 24 enfermeros clínicos que trabajan en departamentos relacionados con el accidente cerebrovascular entre abril y mayo de 2024. Basándose en el modelo COM-B, los datos fueron analizados utilizando el método de análisis deductivo de contenido con el software NVivo12 y codificación manual.

**Resultados:**

El análisis reveló 11 temas y 22 subtemas asociados con los cuatro dominios del modelo COM-B, incluyendo 15 barreras y 7 facilitadores.

**Conclusión:**

La adherencia de los enfermeros clínicos a la correcta posición de las extremidades es dinámica y el posicionamiento debe abordar barreras relacionadas con la capacidad, oportunidad y motivación, tales como priorizar la formación en conocimientos y habilidades para los enfermeros clínicos, mejorar el cumplimiento entre pacientes y cuidadores, establecer sistemas y mecanismos de supervisión robustos, y apoyar la investigación científica e innovación para explorar soluciones de gestión inteligente.

## INTRODUCCIÓN

El accidente cerebrovascular (ACV) es un problema de salud pública importante a nivel mundial y una de las principales causas de discapacidad y mortalidad a largo plazo^(1)^. Se estima que más de 11.9 millones de personas en todo el mundo sufren un accidente cerebrovascular, con un estimado de 7.3 millones de muertes. Además, el accidente cerebrovascular representa el 5.6% de todos los años de vida ajustados por discapacidad (AVAD)^(2)^. A nivel mundial, China tiene el riesgo más alto de sufrir un accidente cerebrovascular a lo largo de la vida, lo que se ha convertido en la principal causa de discapacidad y muerte entre los adultos^(2)^. Los datos indican que cada año se producen aproximadamente 3 millones de nuevos casos de accidente cerebrovascular en China, y más del 80% de estos pacientes experimentan diversos grados de disfunción, siendo la manifestación más común la hemiplejía^(3)^, lo que reduce significativamente la independencia, la calidad de vida y la adaptabilidad social de los pacientes, al tiempo que impone un importante estrés mental y una carga económica a las personas, las familias y la sociedad^(4)^. La aplicación oportuna, científica y eficaz de tratamientos de rehabilitación para mejorar los resultados de la recuperación de los pacientes con accidente cerebrovascular sigue siendo un objetivo fundamental de los investigadores y los profesionales sanitarios de todo el mundo.

La rehabilitación temprana es el método más eficaz para reducir la tasa de discapacidad tras un accidente cerebrovascular, tal y como lo confirma la medicina basada en la evidencia^(5,6)^. La colocación adecuada de las extremidades como posición temporal para prevenir o combatir la espasticidad es un componente clave de la rehabilitación temprana y puede tener un impacto significativo en la salud pública^(7)^. Varias directrices enfatizan la importancia de mantener una posición adecuada de las extremidades en pacientes con accidente cerebrovascular^(5,6,8)^. Esto puede promover indirectamente la formación y remodelación de las sinapsis nerviosas, reparar las neuronas dañadas y es de gran valor para reducir las complicaciones de los pacientes, acelerar el proceso de rehabilitación, mejorar la calidad de vida y reducir la carga económica^(6)^. Además, su practicidad y rentabilidad están bien documentadas en directrices clínicas y declaraciones de consenso de expertos^(5,8)^. A pesar del amplio consenso sobre su eficacia, los enfermeros clínicos (EC) no han aplicado de manera sistemática el posicionamiento adecuado de las extremidades en la práctica diaria, lo que podría obstaculizar el progreso de la rehabilitación de los pacientes con accidente cerebrovascular^(9)^. Es necesario seguir investigando para promover la gestión estandarizada del posicionamiento adecuado de las extremidades.

## ANTECEDENTES

Algunos estudiosos señalan que el cumplimiento por parte de los EC puede ser una razón importante que obstaculiza el desarrollo de la posición adecuada de las extremidades^(7,10)^, y es necesario aclarar los factores que influyen en su aplicación. Estudios anteriores han revelado que la tasa de aplicación de la posición adecuada de las extremidades sigue siendo baja, posiblemente debido a factores como el estado civil, la separación lateral de las extremidades hemipléjicas, la proporción entre camas y cuidadores, la posición, el sistema y la estrategia reguladora^(10,11)^. También es necesario mejorar el nivel cognitivo de los pacientes con ACV y de los cuidadores, lo que también refleja la falta de esfuerzos en materia de educación^(12)^. Para abordar estos retos, los investigadores desarrollaron un índice de revisión de enfermería basado en la evidencia y en las directrices de la Universidad Johns Hopkins. Además, analizaron las barreras para la implementación de la posición adecuada de las extremidades^(10)^. Sin embargo, no se centraron en las experiencias de implementación de los profesionales sanitarios desde una perspectiva cualitativa. Cabe señalar que la implementación eficaz de la posición adecuada de las extremidades requiere la colaboración de los profesionales sanitarios, los pacientes y los familiares. Como principales responsables de la implementación y la educación sobre la posición adecuada de las extremidades, los conocimientos, las actitudes y las experiencias de los enfermeros pueden influir en el cumplimiento por parte de los pacientes y sus familias^(13)^. Por lo tanto, la realización de entrevistas en profundidad para explorar las experiencias de los enfermeros con la implementación de la posición adecuada de las extremidades puede proporcionar una visión más profunda, compleja y completa de sus experiencias, actitudes o comportamientos.

Las investigaciones indican que el uso de un marco teórico para comprender el comportamiento es uno de los enfoques más eficaces^(14)^. El modelo Capacidad, Oportunidad, Motivación-Comportamiento (COM-B por su sigla en inglés), propuesto por primera vez por Michie et al.^(15)^, constituye el núcleo de la rueda del cambio de comportamiento (Behavior Change Wheel - BCW). Este modelo está diseñado para identificar los comportamientos objetivo de poblaciones específicas y facilitar el cambio de comportamiento (Figura 1). El modelo COM-B hace hincapié en que la capacidad, la oportunidad y la motivación dentro de un sistema conductual interactúan y se influyen mutuamente. Estas interacciones están determinadas por las capacidades físicas y mentales de cada individuo, las oportunidades ambientales y sociales, y los procesos reflexivos y automáticos, que en última instancia determinan los resultados conductuales. Múltiples estudios han demostrado que el modelo identifica eficazmente las barreras y los facilitadores, proporcionando posibles objetivos para el diseño de intervenciones y sirviendo como un marco sólido para la investigación sobre conductas saludables^(15–17)^.

**Figura 1:** El modelo COM-B^(15)^.

Por lo tanto, este estudio aplica el modelo COM-B para llevar a cabo una exploración cualitativa de los factores que promueven y dificultan la implementación de una posición adecuada de las extremidades por parte de los enfermeros clínicos. El objetivo es identificar los factores influyentes que pueden abordarse para facilitar la estandarización del manejo de la posición de las extremidades y mejorar los resultados de la rehabilitación de los pacientes con ACV.

## MÉTODO

### Diseño del Estudio

Dada la naturaleza exploratoria de la pregunta de investigación, se adoptó un enfoque fenomenológico descriptivo para este estudio^(18)^. Esta metodología es especialmente adecuada para investigaciones que captan las experiencias de los participantes, sobre todo en contextos en los que se sabe poco sobre el fenómeno que se investiga. El informe del estudio se ajustó a los Criterios Consolidados para la Presentación de Informes de Investigación Cualitativa (COREQ) (Archivo S1).

### Población

Para garantizar la representatividad de la muestra, se reclutó a enfermeras y enfermeros certificados de nueve hospitales de Shandong (China) a través de una convocatoria en línea entre abril y mayo de 2024. Se utilizó el muestreo de máxima variación, una forma de muestreo intencional, para seleccionar a las participantes, lo que garantizó una información completa y mejoró la validez externa del estudio^(18)^. Los criterios de inclusión fueron: (1) enfermeras certificadas que trabajaran en departamentos relacionados con los accidentes cerebrovasculares, incluidos neurología, neurocirugía y rehabilitación; (2) posesión de una licencia de enfermería válida; y (3) participación voluntaria con consentimiento por escrito. Los criterios de exclusión fueron: (1) enfermeras certificadas de vacaciones, en traslado, estudios superiores o formación regular; (2) participación en otros ensayos clínicos; y (3) retirada durante el proceso de entrevista. El tamaño de la muestra se determinó por saturación temática. Se invitó a un total de 27 enfermeras certificadas, 2 declinaron por conflictos de agenda y 1 se retiró durante el proceso de entrevista. Finalmente, las entrevistas con 24 enfermera/os lograron la saturación temática.

### Recolección de Datos

Para la recolección de datos se adoptaron entrevistas individuales semiestructuradas, cara a cara y en profundidad. El equipo de investigación elaboró la guía de entrevistas semiestructuradas revisando la bibliografía pertinente, integrando el marco del modelo COM-B y consultando a expertos para finalizar el contenido. Para realizar una prueba previa de los temas y la estructura de las entrevistas, se llevaron a cabo entrevistas piloto con dos enfermeras certificadas antes de comenzar el estudio. El contenido y la comprensibilidad de la guía de entrevistas parecían buenos. Por lo tanto, no se realizaron adaptaciones a la guía de entrevistas. Además, también se probó el funcionamiento de la grabadora de voz y no se encontraron problemas. Todas las entrevistas fueron realizadas por dos investigadores de posgrado formados en técnicas de entrevista en profundidad con experiencia profesional en enfermería de accidentes cerebrovasculares (con experiencia previa en la realización de 15 entrevistas), cuya participación estuvo motivada por observaciones clínicas y el valor de la investigación. Antes de las entrevistas formales, se explicaron a los participantes los objetivos del estudio, los métodos y los protocolos de confidencialidad. Las entrevistas se grabaron en formato de audio y se tomaron notas a lo largo de las mismas, tras obtener el consentimiento informado. Para proteger la privacidad de los participantes, la entrevista se llevó a cabo en una sala tranquila y cómoda, sin nadie más presente excepto los participantes y los investigadores. Durante la entrevista se observaron y registraron las señales verbales y no verbales de los participantes. Cada sesión duró aproximadamente entre 30 y 45 minutos. La recolección de datos continuó hasta alcanzar la saturación temática, operacionalizada como la ausencia de temas nuevos en tres entrevistas sucesivas, tras lo cual se dio por concluido el reclutamiento de participantes.

### Análisis y Tratamiento de los Datos

Se asignaron números de identificación a los encuestados según el orden de sus entrevistas para proteger su privacidad. En un plazo de 12 horas después de cada entrevista, dos investigadores transcribieron de forma independiente las grabaciones de audio a texto y las cotejaron para garantizar la exactitud de los datos. Las ambigüedades se aclararon rápidamente con los entrevistados para mantener la autenticidad del contenido. Los datos se analizaron empleando el método de análisis deductivo de contenido con el software NVivo12 y codificación manual^(19)^. Los datos se prepararon y se analizaron basándose en los tres antecedentes del modelo COM-B (capacidad, oportunidades y motivación). El equipo de investigación desarrolló colectivamente un esquema de codificación basado en el modelo COM-B para garantizar la coherencia de la codificación. Siguiendo este esquema, dos investigadores realizaron de forma independiente la primera ronda de codificación de todas las transcripciones, y se celebraron reuniones semanales de codificación para discutir las discrepancias y llegar a un consenso. Los códigos se clasificaron o ilustraron dentro de los constructos del modelo. Si algún contenido no se ajustaba a los códigos existentes, se generaba un nuevo código que se evaluaba posteriormente durante el proceso de revisión. Se invitó a expertos externos a revisar los resultados de la codificación. Tras repetidas revisiones de las entradas y los códigos, se resumieron las categorías y los temas finales desde una perspectiva global, y se interpretaron los resultados en consecuencia.

### Aspectos Éticos

Esta investigación se ajusta a las normas éticas establecidas en la Declaración de Helsinki y el Código de Conducta para la Integridad Científica de los Países Bajos^(20)^. El protocolo del estudio fue aprobado por el Comité de Ética (n.º de aprobación SWYX: 2024-029). Todos los participantes firmaron un consentimiento informado por escrito y todos los datos recolectados se utilizaron exclusivamente para este estudio.

## RESULTADOS

Participaron en entrevistas semiestructuradas y presenciales un total de 24 enfermeras/os registradas de entre 25 y 47 años. La mayoría de los enfermeros trabajaban en centros hospitalarios terciarios (n = 13, 54,2%) y la mayoría eran mujeres (n = 23, 95,8%). Su experiencia clínica oscilaba entre 1 y 29 años. Los detalles de las características de los enfermeros se presentan en la Tabla 1.

**Tabla 1:** Datos demográficos de los participantes – Shandong, China, 2024.

Característica	Total (n = 24)
Género	
Hombre	1 (4,2%)
Mujer	23 (95,8%)
Edad (x ± s)	34,38 ± 6,96
Nivel hospitalario	
Hospital secundario	7 (29,2%)
Hospital terciario	17 (70,8%)
Departamento	
Neurología	17 (70,8%)
Departamento de Neurociencias	3 (12,5%)
Cirugía militar	2 (8,3%)
Departamento de Medicina de Rehabilitación	2 (8,3%)
Formación académica	
Grado	23 (95,8%)
Posgrado	1 (4,2%)
Años de experiencia laboral (x ± s)	12,02 ± 7,77
Categorías y títulos profesionales	
Enfermera	2 (8,3%)
Enfermera principal	8 (33,3%)
Enfermera responsable	11 (45,8%)
Subdirectora de enfermería	3 (12,5%)
Puesto	
Enfermera/o	21 (87,5%)
Jefa de enfermería	3 (12,5%)

Los enfermeros clínicos (EC) expresaron un fuerte deseo de implementar una posición adecuada de las extremidades; sin embargo, su cumplimiento se vio influido por múltiples factores. El análisis reveló 11 temas relacionados con los cuatro ámbitos del modelo COM-B y 22 subtemas, entre los que se incluyen 15 barreras y 7 facilitadores. Estos temas están estrechamente alineados con los componentes básicos del modelo COM-B y se detallan en la Tabla 2.

**Tabla 2:** Barreras y facilitadores identificados en los distintos ámbitos del COM-B – Shandong, China, 2024.

COM-B	Temas	Subtemas	Barreras/Facilitadores
Capacidades	El estado físico de los pacientes	Estado de la enfermedad	Barreras
		Pacientes especiales	Barreras
	Conocimientos, habilidades y accesibilidad a la información	Falta de conocimientos y habilidades	Barreras
		El efecto de la formación no es bueno	Barreras
	Cumplimiento por parte del paciente y la familia	Los niveles cognitivos son limitados	Barreras
		Cambios en la forma de educar	Facilitadores
		Características demográficas	Barreras
Oportunidad	Disponibilidad de tiempo	Operación que requiere mucho tiempo	Barreras
		Trabajo de alta intensidad	Barreras
	Medios auxiliares	Escasez de cantidad	Barreras
		Diferencias en la calidad	Barreras
	Asignación y utilización de los recursos médicos y sanitarios	Asignación irrazonable de los recursos humanos	Barreras
		Colaboración y comunicación multidisciplinarias	Facilitadores
		La distribución de los recursos médicos es desigual	Barreras
	Influencia social	Buen ambiente de trabajo	Facilitadores
Motivación	El sistema y la estrategia de supervisión adecuados	Falta de criterios de evaluación	Barreras
		Falta de estrategia reguladora	Barreras
	Creencias sobre las consecuencias	El efecto positivo de la colocación adecuada de las extremidades	Facilitadores
		Comentarios positivos	Facilitadores
	Directrices y políticas relacionadas con la posición adecuada de las extremidades	Faltan directrices y políticas que orienten la práctica de la posición adecuada de las extremidades	Barreras
	Sentido de la valía profesional	Sentido del logro en el trabajo	Facilitadores
		Ética laboral	Facilitadores

### Capacidades

#### El Estado Físico de los Pacientes

Muchos EC informaron que, aunque estaban dispuestos a colocar las extremidades en la posición adecuada, los pacientes a menudo se irritaban o se sentían incómodos, lo que dificultaba mantener la postura deseada. A veces esto era imposible debido al tono muscular elevado y la rigidez de las extremidades. «El paciente está irritable o confuso, por lo que puede recuperar su estado después de un rato» (EC1).

Además, los EC señalaron que la aplicación de una posición adecuada de las extremidades en pacientes con mucho peso o úlceras por presión planteaba considerables retos físicos, humanos y materiales. «Si el paciente pesa demasiado, será difícil» (EC12).

Por lo tanto, la dificultad de aplicar una posición adecuada de las extremidades aumenta considerablemente debido a factores específicos del paciente y se ve agravada por las limitaciones de los recursos de enfermería.

#### Conocimientos, Habilidades y Accesibilidad de la Información

Lamentablemente, muchos EC expresaron incertidumbre sobre el momento y los principios de la colocación adecuada de las extremidades. «No hay tiempo y el principio de colocación no está muy claro» (EC1). Las enfermeras con menos experiencia laboral informaron de mayores dificultades debido a la falta de conocimientos. «Soy una enfermera junior y me resulta un poco difícil contactar con ellos» (EC4).

Por el contrario, poseer los conocimientos y habilidades adecuados es un factor facilitador. Por lo tanto, varios EC hicieron hincapié en la necesidad de mejorar los métodos de formación y el acceso a la información. «Se recomienda que la formación combine teoría y práctica para que tenga más claro cómo hacerlo la próxima vez que se lo presente a los pacientes» (EC10).

Así pues, la competencia profesional insuficiente limita la aplicación estandarizada de la colocación adecuada de las extremidades, y la formación deficiente afecta a la accesibilidad de la información.

#### Cumplimiento Por Parte del Paciente y la Familia

Todos los EC hicieron hincapié en que el posicionamiento adecuado de las extremidades requiere la cooperación de los pacientes, los cuidadores y el personal de enfermería. Sin embargo, muchos pacientes y cuidadores no reconocen la importancia del posicionamiento adecuado de las extremidades e incluso pueden expresar escepticismo debido a la falta de resultados visibles inmediatos. «Los cuidadores no entienden por qué lo hacen, ya que no ven ninguna diferencia clara» (EC1).

La educación sanitaria desempeña un papel fundamental en la mejora de los niveles cognitivos de los pacientes y los cuidadores. Muchos EC informaron haber adoptado métodos educativos innovadores, con resultados prometedores. «Hemos creado un código QR para convertir el contenido de la propaganda en imágenes o vídeos animados, lo que resulta más cómodo e intuitivo» (EC20). «Organizamos conferencias y concursos con regularidad, y los resultados son buenos» (EC15).

Las características de los pacientes y cuidadores, como la edad, el nivel educativo y la paciencia, se identificaron como posibles barreras para una transmisión eficaz de los conocimientos. Esto pone de relieve la importancia de que los enfermeros dominen las habilidades de comunicación para mejorar la comprensión. «Estoy cansada de enseñar a personas mayores porque les cuesta aprender» (EC11).

Por lo tanto, se puede observar que el nivel cognitivo, los métodos educativos y las características demográficas influyen en el grado de cooperación de los pacientes y sus familias con el tratamiento.

### Oportunidad

#### Disponibilidad de Tiempo

La disponibilidad de tiempo supuso un reto importante. Muchos EC señalaron que mantener la posición adecuada de las extremidades requiere girar a los pacientes cada dos horas. Dado que los pacientes a menudo no pueden mantener la postura correcta, es necesario supervisarlos y ajustarlos constantemente, lo que requiere mucho tiempo y energía. «Creo que la implementación es demasiado complicada. Es imposible ajustarlo todo el tiempo» (EC2).

Además, la alta intensidad del trabajo agrava aún más los retos a los que se enfrentan los EC. «Los pacientes con ACV son más graves, con más operaciones de enfermería y cuidados básicos» (EC16). Algunos EC sugirieron que la integración de herramientas o tecnología inteligentes podría mejorar el proceso. «Si existiera un método inteligente para resolverlo, la implementación de la posición adecuada de las extremidades no sería tan problemática» (EC1).

Por lo tanto, la complejidad de la operación y la presión del trabajo han intensificado el consumo de tiempo y energía. Para resolver este problema, los EC sugieren utilizar tecnologías inteligentes para ayudar en la gestión.

#### Medios Auxiliares

A menudo se utilizan múltiples almohadas y otras herramientas para sostener el cuerpo del paciente. Sin embargo, la falta de herramientas auxiliares adecuadas afecta significativamente a la implementación de una posición adecuada de las extremidades. «Es posible que los cuidadores no puedan comprar almohadas; la sala no tiene muchas, solo puede proporcionar una almohada a los pacientes» (EC24). «Espero que el hospital aporte algunos fondos y proporcione más herramientas auxiliares» (EC8).

Además, la calidad de las herramientas auxiliares también afecta a su eficacia. Problemas como el apoyo insuficiente tras un uso prolongado, los materiales incómodos y la imposibilidad de ajustar la altura o los ángulos plantean dificultades. «El paciente se acuesta, la almohada no alcanza esa altura, la almohada giratoria es un poco dura, lo que es malo para la piel del paciente» (EC23).

Por lo tanto, la cantidad insuficiente y los defectos de calidad de las herramientas de asistencia dificultan la implementación de una posición adecuada de las extremidades e incluso pueden tener efectos adversos para los pacientes.

#### Asignación y Utilización de los Recursos Médicos y Sanitarios

Muchos EC informaron de que la asignación irrazonable de los recursos humanos supone un obstáculo importante para la posición adecuada de las extremidades. «Hay entre 80 y 90 pacientes en nuestro departamento, pero solo 15 enfermeras, por lo que la falta de personal es un problema que no se puede ignorar» (EC13).

Para hacer frente a estos retos, algunos EC han formado equipos de tratamiento multidisciplinario (MDT), que reducen la carga de trabajo y trascienden las fronteras profesionales, fomentando la confianza entre médicos y pacientes. «Los médicos dan más importancia a las posiciones funcionales y nos orientan más» (EC7). «Cuando hablamos, no tenemos tanto peso como los médicos, y los pacientes creen más en ellos» (EC24).

La falta de énfasis en la posición adecuada de las extremidades en los hospitales de atención primaria repercute directamente en el pronóstico de los pacientes, una preocupación expresada con frecuencia por los enfermeros. «Lamentamos que los pacientes de los hospitales básicos tengan pies caídos que no se pueden corregir» (EC15). Para mitigar este problema, los enfermeros sugirieron reforzar la colaboración y la comunicación entre los hospitales. «Creo que necesito ir al hospital superior a estudiar, allí puedo ver más» (EC3).

Así, la escasez de recursos humanos y la insuficiencia de los recursos de base han obstaculizado la estandarización de la atención, mientras que la cooperación multidisciplinar e interhospitalaria puede ayudar a paliar este problema.

#### Influencia Social

La dinámica social de los individuos y los grupos puede influir en las actitudes y comportamientos de los demás en direcciones específicas. Varios EC destacaron que un ambiente de trabajo positivo les motiva a implementar el posicionamiento adecuado de las extremidades. «Nos comunicamos bien con los pacientes y los cuidadores, y también estamos dispuestos a educarlos» (EC19). «No lo hacemos solos, sino que todo el departamento lo hace, por lo que es más fácil implementarlo» (EC4).

Por lo tanto, una cultura de equipo positiva y la comunicación entre médicos y pacientes pueden influir positivamente en los comportamientos de enfermería y reducir la dificultad de implementar el posicionamiento adecuado de las extremidades.

### Motivación

#### El Sistema y la Estrategia de Supervisión Pertinentes

Muchos EC informaron de la falta de criterios de evaluación clínicamente disponibles para determinar el buen posicionamiento de las extremidades, lo que dificulta la evaluación de la eficacia de la implementación. «El estándar de evaluación para el posicionamiento adecuado de las extremidades depende principalmente de la recuperación funcional intuitiva» (EC18).

Además, varios EC observaron que los hospitales y los departamentos no son estrictos en la implementación de las extremidades adecuadas. Por el contrario, la atención de los líderes mejora significativamente el cumplimiento. «Aunque se inspecciona dos veces por semana, no parece que se haya implementado, por lo que la supervisión del departamento puede ser un poco mayor» (EC10).

Por lo tanto, la falta de criterios de evaluación y de una estrategia reguladora ha reducido el énfasis y la capacidad de implementación de los EC en el posicionamiento correcto de las extremidades, lo cual es un factor objetivo importante.

#### Creencia Sobre las Consecuencias

Todos los EC coincidieron en que el posicionamiento adecuado de las extremidades tiene un impacto positivo en los pacientes, las familias y la sociedad, al tiempo que reduce en cierta medida la carga de trabajo de los enfermeros. «La intervención temprana puede favorecer la recuperación de la función de las extremidades del paciente y prevenir algunas complicaciones» (EC4). «Si se producen úlceras por presión, aumentará nuestra carga de trabajo» (EEC19). Desde la perspectiva del paciente, los EC señalaron que el posicionamiento adecuado de las extremidades es una intervención relativamente sencilla y rentable. «Enseñe a los cuidadores cómo hacerlo; también pueden hacerlo en casa» (EEC6).

Además, los comentarios positivos de los pacientes y los cuidadores, así como la eficacia observable, motivan aún más el impulso interno para convencerse de los beneficios del posicionamiento adecuado de las extremidades. «El edema de las extremidades de algunos pacientes se ha reducido significativamente, lo que indica que la implementación ha sido eficaz» (EC11).

Los EC reconocen en general los beneficios mutuos de la posición adecuada de las extremidades, y los comentarios positivos refuerzan aún más su confianza en los resultados, creando un círculo virtuoso.

#### Directrices y Políticas Relacionadas con la Posición Adecuada de las Extremidades

Varios EC expresaron su deseo de adoptar prácticas más estandarizadas, pero destacaron la ausencia de directrices autorizadas o de consenso de expertos que orienten la implementación clínica. «Realmente queremos llevar a cabo el posicionamiento adecuado de las extremidades, pero no hemos encontrado el índice publicado por el Estado» (EC3).

En cuanto a las políticas, los EC hicieron hincapié en la necesidad de que se prestara más atención a nivel nacional al desarrollo del sistema de atención primaria. «Que los hospitales básicos aprendan más, mejoren la concienciación sobre el buen posicionamiento de las extremidades» (EC8).

De ello se desprende que la falta de directrices autorizadas y de apoyo político ha limitado el desarrollo de la capacidad profesional de los EC y ha debilitado su motivación práctica.

#### Sentido de su Valor Profesional

Varios EC señalaron que los resultados positivos de los pacientes refuerzan el valor y la importancia de sus esfuerzos. «Los pacientes con una estancia hospitalaria prolongada, sin contracturas o con una mejor recuperación, tienen una gran sensación de logro» (EC1).

Además, la ética profesional desempeña un papel fundamental en su compromiso con la colocación adecuada de las extremidades. «El concepto y el pensamiento han formado la inercia del pensamiento. Si un paciente tiene hemiplejía, hay que guiarlo y ayudarlo con la rehabilitación funcional» (EC8).

Así, los logros profesionales y la ética laboral refuerzan el reconocimiento del valor profesional de los EC, lo que sirve de motor para la práctica de enfermería estandarizada.

Estos hallazgos revelan que la implementación de la posición adecuada de las extremidades se enfrenta a retos interconectados relacionados con los pacientes (barreras físicas/cognitivas), los enfermeros (conocimientos/limitaciones de tiempo) y el sistema (recursos/formación). Si bien algunos factores relacionados con los pacientes son inherentes, existen barreras modificables, como la formación, la educación sanitaria y la mejora de los equipos, que ofrecen soluciones viables, siendo la tecnología y la educación innovadora especialmente prometedoras. La interdependencia de estos obstáculos pone de relieve la necesidad de intervenciones integradas que aborden la capacidad, la oportunidad y la motivación para impulsar un cambio de comportamiento sostenible.

## DISCUSIÓN

Este estudio identificó 11 temas que se alineaban perfectamente con el modelo COM-B, lo que ofrece una comprensión integral de los factores que influyen en la implementación de la posición adecuada de las extremidades por parte de los enfermeros^(15)^. Los hallazgos sugieren que el cumplimiento de la posición adecuada de las extremidades por parte de los enfermeros es un proceso dinámico y equilibrado influenciado por su capacidad, oportunidad y motivación.

Para una práctica eficaz, era esencial contar con conocimientos y habilidades sólidos^(10,12)^. Sin embargo, en el presente estudio, los enfermeros parecían considerar que el momento y la implementación del posicionamiento adecuado de las extremidades eran problemas completamente independientes, y algunas lo percibían como algo ajeno a su ámbito profesional. La confianza y la experiencia derivadas de conocimientos y habilidades sólidos desempeñan un papel crucial en la promoción de la estandarización del posicionamiento adecuado de las extremidades^(7,21)^. En consecuencia, los enfermeros clínicos de este estudio expresaron una gran necesidad de formación para adquirir conocimientos y habilidades relacionados con la posición adecuada de las extremidades, lo que sugiere que los métodos de formación tradicionales son cada vez más inadecuados para satisfacer sus necesidades de aprendizaje^(22)^. Dado el rigor que exige la práctica médica, se espera que la formación basada en la evidencia mejore las actitudes, los conocimientos, las habilidades, la autoeficacia y el comportamiento profesional de los enfermeros^(23)^. Estas intervenciones también deben hacer hincapié en la importancia de desarrollar directrices exhaustivas y un consenso de expertos sobre la posición de las extremidades. Como destacan las EC, incluso con recursos limitados, el acceso a directrices o al consenso de expertos puede mejorar su motivación intrínseca, promoviendo así el cumplimiento de las prácticas adecuadas de posicionamiento de las extremidades.

El grado de alineación entre los comportamientos de los pacientes y los cuidadores en la búsqueda de la recuperación y el cumplimiento de los consejos médicos influye significativamente en las actitudes de las EC hacia su papel en la atención al paciente, en consonancia con investigaciones anteriores^(24)^. Comprender la importancia de la posición adecuada de las extremidades y su papel en la recuperación es esencial para fomentar una actitud positiva hacia la participación en la toma de decisiones^(25)^. Esta brecha en el conocimiento y la confianza es una razón fundamental del escepticismo observado entre los pacientes y sus familias en este estudio. Varias directrices enfatizan la necesidad de que los profesionales sanitarios den prioridad a la educación sobre la rehabilitación de los pacientes con ACV^(5,6)^. Aunque se han llevado a cabo con éxito reformas en la prestación de servicios de enfermería inteligente y educación sanitaria, aún se enfrentan múltiples retos, como la brecha digital, la sobrecarga de información, la personalización insuficiente y la seguridad de la privacidad^(26)^. Además de los problemas de cumplimiento, factores como el estado del paciente, su peso y las complicaciones plantean retos a los EC. Para los pacientes que se enfrentan a estas barreras, es importante explorar estrategias óptimas para la rehabilitación temprana y la posición adecuada de las extremidades.

Aunque el posicionamiento adecuado de las extremidades ofrece numerosas ventajas, su persistencia, su naturaleza detallada y sus tediosos procesos requieren mucho tiempo, mano de obra, recursos materiales y esfuerzo físico^(9,11)^. Estas exigencias, especialmente en entornos de trabajo de alta intensidad, la naturaleza fragmentada, caótica y no lineal del trabajo de enfermería, junto con el desarrollo de alta calidad de los hospitales públicos y la disminución de la duración media de las estancias hospitalarias, complican la capacidad de los EC para dar prioridad a las prácticas de posicionamiento de las extremidades de los pacientes^(27)^. La asignación irracional de los recursos de salud agrava este desafío, como la proporción entre camas y cuidadores y la distribución desigual de los recursos médicos entre las zonas urbanas y rurales^(28)^. Para abordar estos problemas, algunos investigadores han desarrollado modelos de asignación de recursos humanos basados en las horas de enfermería o en índices de casuística, y han establecido alianzas médicas regionales y equipos de colaboración multidisciplinarios para optimizar e integrar los recursos médicos^(29)^. Estas ideas sugieren que tanto el Estado como los hospitales deben trabajar activamente para avanzar en la modernización de la capacidad con el fin de abordar de manera fundamental los retos en materia de recursos. Además, un ambiente de trabajo positivo puede animar a los EC a autorregularse y adoptar las mejores prácticas, lo que se atribuye al hecho de que los pensamientos, sentimientos, actitudes y comportamientos de las personas suelen estar influenciados por las palabras y acciones de los demás(25).

La implementación de una posición adecuada de las extremidades se ve notablemente influenciada por la disponibilidad y la calidad de los soportes auxiliares, lo que representa una barrera significativa en consonancia con los resultados de estudios previos^(10,11)^. En la práctica clínica actual, los cuidadores y el personal de enfermería dependen de ropa de cama, almohadas, almohadas giratorias y otras herramientas para ayudar a los pacientes a ajustarse. Sin embargo, los problemas relacionados con la cantidad y la calidad de estos soportes a menudo comprometen la eficacia de la posición adecuada de las extremidades^(11)^. La disponibilidad insuficiente de apoyos auxiliares puede deberse a las limitaciones presupuestarias de los hospitales, el entorno y la situación económica o la intención de compra de los pacientes y los cuidadores^(9)^. Además, debe tenerse en cuenta la calidad de los apoyos auxiliares. El material, el tamaño y el ángulo de los apoyos auxiliares influyen significativamente en la aplicación eficaz de la posición adecuada de las extremidades e incluso pueden suponer un riesgo para la salud de los pacientes^(11)^. Algunos ejemplos son el dolor de hombro causado por una posición incorrecta de las extremidades y las lesiones por presión cutánea resultantes de la fuerza bruta. Estos ejemplos ponen de relieve la importancia de mejorar los sistemas de apoyo auxiliar. La enfermería inteligente ofrece nuevas oportunidades para la gestión inteligente de la posición adecuada de las extremidades. El aprovechamiento de la tecnología de inteligencia artificial para desarrollar dispositivos de gestión inteligente podría resolver las limitaciones de los soportes auxiliares tradicionales, lo que representa un área clave de interés para futuras investigaciones.

Por último, en el ámbito de la motivación, los EC expresaron un fuerte reconocimiento y aprecio por la pronta implementación del posicionamiento adecuado de las extremidades, destacándolo como un factor clave para promover su desarrollo^(16,17)^. Mientras tanto, los comentarios positivos de los pacientes, los cuidadores y los resultados de la implementación refuerzan aún más su motivación. Además, la sensación de logro profesional y la adhesión a los principios éticos contribuyen a su autoeficacia, lo que les permite superar ciertos retos en la práctica del posicionamiento adecuado de las extremidades. Sin embargo, la motivación se ve inevitablemente influida por factores objetivos, como la falta de criterios de evaluación estandarizados y de mecanismos reguladores, que pueden influir en el ajuste del comportamiento y desviarlo de las expectativas^(10)^. Por lo tanto, es necesario mejorar los incentivos y mantener el desarrollo del comportamiento de los EC.

El examen de los datos sociodemográficos respalda aún más los resultados de este estudio: los EC avanzados (≥10 años de experiencia, n = 16/24) dan prioridad a las cuestiones a nivel del sistema, mientras que los EC junior (≤3 años, n = 5/24) se centran en las habilidades, lo que concuerda con la teoría novus-expert de Benner^(30)^. Los EC de los hospitales terciarios muestran una mayor familiaridad con los procesos que sus homólogos de los hospitales secundarios, lo que pone de relieve que las diferencias en los recursos de formación pueden afectar al desarrollo de capacidades. Todo ello pone de relieve la interacción dinámica entre las capacidades, las oportunidades y la motivación en la transformación del comportamiento. Cabe señalar que la insuficiencia de la muestra de EC masculinos (4,2%) puede afectar al análisis de las diferencias de género, por lo que se optimizará en futuros estudios.

### Limitaciones

Este estudio tiene varias limitaciones. En primer lugar, la fenomenología hace hincapié en el papel interpretativo de los investigadores y puede verse influida por sus experiencias y valores, lo que da lugar a sesgos subjetivos. En segundo lugar, el pequeño tamaño de la muestra, típico de los estudios fenomenológicos, aunque permite una exploración en profundidad, puede limitar la generalización de los resultados a poblaciones más amplias. En tercer lugar, este estudio se limitó a las perspectivas de los enfermeros neuro-especializados. La incorporación de las perspectivas de los pacientes, los cuidadores y los médicos podría proporcionar una comprensión más amplia. Por último, la transferibilidad de los hallazgos a los EC fuera de China puede ser limitada debido a las variaciones en las funciones y responsabilidades de los EC en los distintos países, lo que requiere una mayor exploración.

## CONCLUSIÓN

Los resultados indican que la adherencia de los EC al posicionamiento adecuado de las extremidades es dinámica y que el posicionamiento debe abordar las barreras relacionadas con la capacidad, la oportunidad y la motivación. Esto subraya la importancia de mejorar la formación de los EC en materia de conocimientos y habilidades para el posicionamiento de las extremidades, ya que estos constituyen la base de la práctica profesional. Dicha formación debe complementarse con esfuerzos para mejorar el cumplimiento entre los pacientes y sus familias. Además, el Estado y las instituciones sanitarias deben dar prioridad a la asignación y el uso racional de los recursos médicos, lo que incluye optimizar la asignación de recursos, promover modelos de colaboración multidisciplinaria, establecer mecanismos de monitoreo y evaluación, llevar a cabo investigación científica e innovación, etc., con el fin de estandarizar las prácticas adecuadas de posicionamiento de las extremidades y mejorar los resultados de los pacientes.

### Implicaciones

Los conocimientos adquiridos en este estudio pueden servir de base para el desarrollo de programas de formación y estrategias de implementación adaptados a los EC, al tiempo que proporcionan orientación para la transformación inteligente de la gestión adecuada del posicionamiento de las extremidades. Al mejorar su capacidad de práctica profesional e innovar los procesos de flujo de trabajo, estas iniciativas mejorarán en última instancia los resultados de la rehabilitación y el pronóstico de los pacientes con ACV.

## Data Availability

El conjunto completo de datos que respalda los hallazgos de este estudio está disponible solicitándolo al autor correspondiente [Xia Chen]. Los datos no están disponibles públicamente debido a que contienen información que compromete la privacidad de los participantes de la investigación.

## SUPPLEMENTARY MATERIAL

El siguiente material suplementario está disponible en línea:

File S1 – PRISMA 2020 Checklist.
